# Fluctuation Scaling, Taylor’s Law, and Crime

**DOI:** 10.1371/journal.pone.0109004

**Published:** 2014-10-01

**Authors:** Quentin S. Hanley, Suniya Khatun, Amal Yosef, Rachel-May Dyer

**Affiliations:** School of Science and Technology, Nottingham Trent University, Nottingham, United Kingdom; Arizona State University, United States of America

## Abstract

Fluctuation scaling relationships have been observed in a wide range of processes ranging from internet router traffic to measles cases. Taylor’s law is one such scaling relationship and has been widely applied in ecology to understand communities including trees, birds, human populations, and insects. We show that monthly crime reports in the UK show complex fluctuation scaling which can be approximated by Taylor’s law relationships corresponding to local policing neighborhoods and larger regional and countrywide scales. Regression models applied to local scale data from Derbyshire and Nottinghamshire found that different categories of crime exhibited different scaling exponents with no significant difference between the two regions. On this scale, violence reports were close to a Poisson distribution (*α* = 1.057±0.026) while burglary exhibited a greater exponent (*α* = 1.292±0.029) indicative of temporal clustering. These two regions exhibited significantly different pre-exponential factors for the categories of anti-social behavior and burglary indicating that local variations in crime reports can be assessed using fluctuation scaling methods. At regional and countrywide scales, all categories exhibited scaling behavior indicative of temporal clustering evidenced by Taylor’s law exponents from 1.43±0.12 (Drugs) to 2.094±0081 (Other Crimes). Investigating crime behavior via fluctuation scaling gives insight beyond that of raw numbers and is unique in reporting on all processes contributing to the observed variance and is either robust to or exhibits signs of many types of data manipulation.

## Introduction

The statistics of crime, criminals, and criminal justice systems have been studied by scientists and mathematicians since Poisson investigated the French Jury System [1]. Scaling behavior of crime with populations has been studied in many parts of the world [2], [3], [4], [5]. In these contexts, it has been noted that the scaling of crime, particularly violence, varies in different countries and regions. It has been noted that in the case of the US murder rate, the number of murders appears to be nearly a Poisson process with a scaling factor of 1.04 [1], [6] indicating that crime may be amenable to a fluctuation scaling analysis.

The science of fluctuation scaling began with optimization of agricultural yields where it was noted that a plot of the logarithm of yield variance on log area produced a linear relationship [7]. Taylor [8], [9] noted a mean-variance plot of populations followed a power law across many species. This relationship became known as Taylor’s Law (TL) and has been widely observed in ecology [10], [11], [12]. A TL relationship has been reported for Measles cases in the UK and the scaling was observed to change as the extent of vaccination increased [13]. Related scaling relationships have been observed outside of biology including such things as human interactions, stock trades, measures of firm size, and urban automobile traffic [14], [15], [16], [17], [18]. The origin and interpretation of the TL relationships has remained of interest over time, with particular effort directed to understanding the meaning of the exponent and the underlying mathematics [16], [19], [20], [21], [22]. Depending on the system studied, the size of the exponent and the model applied, it has been interpreted to indicate synchronization [17], randomness and aggregation [8], species interaction [22], and multiplicative population growth [19].

In the context of ecology and many other studies concerned with scaling relationships, the focus has been on the exponent rather than interpretation of the pre-exponential factor. The pre-exponential is normally considered a characteristic of the experiment and its associated sampling scheme [9]. However, using concepts from statistical optics the behavior of a particular data set following application of gain via multiplication or division can be determined. The method of mean-variance used to calibrate the gain of charge coupled devices (CCDs) described by Mortara and Fowler relies on the Poisson statistics of photons and is widely used to characterize the gain of imaging detectors [23], [24]. The mean-variance plot reveals the number of photons per arbitrary unit of readout allowing light intensity to be compared across systems and gain settings. In the limit of zero CCD readout noise, a mean-variance plot gives a TL relationship with an exponent of 1.

Here, fluctuation scaling is applied to crime and crime statistics and its potential for testing policing and justice strategies articulated. Using publicly available statistics we show: i) temporal fluctuations in crime follow TL on local and regional scales, ii) exponents vary across crime types, iii) pre-exponential factors vary between jurisdictions, iv) over wider spatial scales crime shows high levels of temporal clustering with some exponents approaching 2; and v) the number of crime reports required to observe larger scale behavior varies for different crimes. To compare to a data set without the controversy of police reported crime, monthly mortality reports for England and Wales were analyzed. We propose that before an argument for a reduction in crime can be confirmed, there must be both a reduction in the number of crimes and a change in the scaling law as has been seen in epidemiology [13]. In the absence of a change in the scaling law, the underlying processes leading to crime cannot be said to have changed. The scaling laws show evidence of or are resistant to many types of data manipulation such as, systematic under-reporting by a factor, stochastic manipulation of data by adding or removing crimes from a data base, working to numerical targets, etc. We anticipate this approach to the use of crime reports and police statistics will become a routine addition to approaches based on the number of crimes and per capita scaling to allow more direct comparisons across nations, states, and regions with similar laws.

### Theory

Taylor’s law is an empirical relationship in which the variance of a system is a power law function of the mean value.
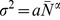
(1)


In this expression, 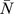 is the mean number of occurrences of a particular type, 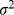 is the variance, *a* is a pre-exponential factor and *α* is an exponent. In the classic treatments in ecology, TL is evaluated at fixed quadrat size. More generally, both *a* and *α* vary with quadrat size and limiting values of *α* have been shown to depend on the extent of randomness within a particular model [25], [26]. As spatial and temporal scales vary, *α* tends to approach limiting values [16]. Here, 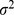 represents temporal fluctuations in the number of events occurring in one month periods computed over 12 months.

In the form given in equation 1, we assume the TL relationship reflects the behavior of the process underlying the events which are sampled and detected perfectly. Experimental pre-exponential factors can provide information about the detector and the process of detection. One example is the determination of multiplicative gain, *G*, applied to a system [23], [24]. A perfect Poisson process (*α* = *a = *1) will produce a mean-variance plot with a slope of one (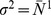). If the mean variance plot of a process known to be Poisson exhibits a slope ≠ 1, this represents gain (e.g. 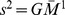, where 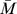 is the average measured signal). Previously this has been applied to photon detectors based on the assumption that photons are Poisson distributed [23], [24]. The approach may be generalized to all power law relationships of the form of equation 1. If the magnitude of the observed events, *M*, is related to the actual events by a gain factor, *G*, such that 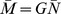 and 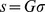, then substitution into equation 1 and rearranging yields:
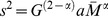
(2)


This expression indicates that as *α* approaches 2, the scaling law becomes gain invariant (e.g.: 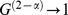 as 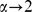) and elsewhere the effect of gain on the power law is predictable with the exponent being invariant to multiplication and division. The invariance of power law exponents to rescaling is well known and equation 2 reinforces prior work such as that arising from the analysis of the spatial and temporal distribution of trees over a period of 70 years using TL and the Lewontin-Cohen model [19]. Equation 2 suggests that in otherwise similar experiments (scale, system, species, etc.) TL relationships having the same *α* and *a* but exhibiting different pre-exponential factors (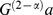) may be interpreted as having different gain. Using experimentally determined TL relationships, gain may be interpreted using two frameworks: absolute and relative *G*.

Absolute *G* comparisons require prior knowledge of both *α* and *a* for the process being studied. If these are known, then *G* may be obtained from equation 2. Gain obtained in this way may be compared directly to any other value of *G* obtained from any TL process and is a characteristic of the detection system not the process being detected.

Relative *G* interpretation requires less prior knowledge but assumes the detection systems report on the same process. For example, consider two constabularies (indexed 1 and 2) reporting crimes of a particular type. Applying equation 2 yields two modified TL relationships corresponding to the two constabularies.
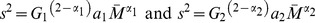
(3)


If statistical tests indicate *α*_1_ = *α*_2_, then apply the single process assumption (e.g.: *α*_1_ = *α*_2_ and *a*_1_ = *a*_2_). This assumption allows the *a*’s to cancel and the index to be dropped from *α* in a ratio of pre-exponential factors, *R*.
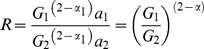
(4)


The relative gain, *G_R,_* can be obtained by rearranging.
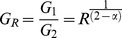
(5)


For clarity, empirically determined pre-exponential factors from TL will be referred to as *A* and assumed to represent the product 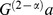. Two points should be noted. First, when the relative gain is one, this means the gains are equal not that gain is absent. Second, the assumption that *α*_1_ = *α*_2_ implies *a*_1_ = *a*_2_ may be challenged but is reasonable for similar events, such as incidents of violence in two adjacent regions. In the absence of this assumption, a discussion of *R* remains valuable as it reports on a combination of G and/or *a*.

The data sets here exhibit TL behavior over limited scales. More generally, the overall fluctuation scaling relationship is described by a function relating the variance to the mean number of observed events.
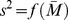
(6)


Since the characteristics of this function may be unknown, we apply TL as an approximation over sections of the fluctuation scaling relationship.

Interpreting fluctuation scaling relationships in the context of crime may be done with a simple model. In the case of perfect policing, justice, rehabilitation and supporting social policies when an individual commits a crime, that individual would be arrested, convicted, jailed, and later released back into the community perfectly reformed with no incentive to re-offend. That offence leads to no further crime and is completely independent of all others. Such true randomness would lead to a fluctuation scaling relationship with an exponent of one. On the other hand, in a different scenario, the individual avoids arrest, re-offends, incites others to crime and changes the community around them leading to clustered crime and scaling laws with exponents other than 1. In the absence of the exact mechanism by which crime becomes clustered, the empirical scaling law will indicate they are not evenly distributed. While there are many ways to explain TL behavior, we will discuss the exponents in terms of apparent randomness and clustering. Apparent randomness will be used to describe *α* = 1 while making clear that models exist in which a Poisson distribution may appear from regularly spaced events [27] and clustering will be used to discuss *α* >1. In the case of crime, it is desirable to develop interventions to detect and disrupt clustering of crime. While it is theoretically possible to have high numbers of apparently random crimes, based on results from epidemiology [13] this is unlikely to occur in response to interventions reducing the TL exponents to 1.

All aspects of the fluctuation scaling experiment (e.g.: the basic underlying events, classification, and reporting) contribute to the system variance. As a result, examination of fluctuation scaling behavior allows a number of questions to be answered: do crime and death appear random, do all crimes exhibit the same scaling behavior, are they scale invariant, if they are not scale invariant does this become apparent at the same scale for all types of crime, and can manipulation of a data set be detected?

## Materials and Methods

### Data Sets

Population data for Nottinghamshire and Derbyshire were obtained from the UK Office of National Statistics (release date 19/12/2013) [28]. Summary crime data for the two regions were obtained from the UK Office of National Statistics (release date 23/1/2014) [29]. Local scale police statistics from the Derbyshire and Nottinghamshire regions were obtained from the UK Home Office via its web site using the policing neighborhoods (accessed between November 2013 and July 2014) [30], [31]. Additional regional and country scale data were obtained from the Economic Policy Centre via its UKCrimeStats web site (access date 27/03/2014) [32]. Mortality data for 2013 were provided by the Office of National Statistics via the report on Monthly Provisional Figures on Deaths Registered in England and Wales (release date 28/01/2014) [33]. All data were used directly. Crime reports provided by the police have been subject to controversy [34]; however, the presence or absence of any manipulation of the data sets was assumed to contribute to the observed variance. Under-reporting has been considered in the context of fluctuation scaling in epidemiology [13] and certain types of manipulation were modeled here. The mortality data are provided in Data File S1 under the terms of the UK Crown Copyright Open Government License. The local data set as collated for this study has been provided in Data File S2 and are provided under the UK Open Government License. The regional scale data have been provided in Data File S3 with permission from the Economic Policy Centre.

### Overview of Regions

The regional and country statistics used here include: England, Wales, and Northern Ireland in the case of Crime and include England and Wales in the case of mortality. Regional mortality statistics were divided by county and unitary authorities aggregated into country segments (e.g. East Midlands, etc.) and then to country level. Policing in England, Wales, and Northern Ireland for the purposes of the crime statistics reported here was organized into 43 constabularies. Each constabulary is broken into policing neighborhoods which vary in size and in the number of crimes typically reported in a one month period. The data provided are subject to change after they first appear and in our experience have changed after a year or more online. This means the data used here represent a snapshot of the time they were accessed. Local scale statistics consisted of data from 151 policing neighborhoods within the two constabularies responsible for Nottinghamshire and Derbyshire. The crimes considered at local scale were: Anti-Social Behavior, Burglary, Violence, and Total Crime. Nottinghamshire and Derbyshire are similar in size (2235 km^2^ and 2703 km^2^, respectively) and population (1,090,700 and 1,039,600, respectively). Nottinghamshire had a higher number of reported crimes (69,277, excluding anti-social behavior) when compared to Derbyshire (52,022, excluding anti-social behavior) for the year ending September 2013 [29]. Regional and country scale data were from 43 Constabularies and considered Anti-Social Behavior, Burglary, Robbery, Criminal Damage and Arson, Violence, Drugs, and Other. The City of London was included in the UK wide aggregated crime reports but omitted from the regional and country scale analysis due its scale being more comparable to a local crime neighborhood than the other constabularies.

### Statistical Analysis

Averages and variances were computed on events reported in one month intervals within a particular location or region over a 12 month period beginning November 2012 and ending October 2013. Data for single crime categories were analyzed by regression of log transformed means and variances by standard least squares. Analysis of local scale data across all crime types and the two counties (Nottinghamshire and Derbyshire) was done using general regression analysis applied to the log transformed mean and variance values with categorical variables using commercially available software (Minitab version 16.2; Minitab Inc.). Data categories were location (0 ≡ Derbyshire and 1 ≡ Nottinghamshire) and type of crime (0 ≡ Total crime, 1 ≡ anti-social-behavior, 2 ≡ burglary, and 3 ≡ violence) and these were analyzed including all variables and interactions (e.g.: location, crime, log(mean), location*crime, location*log(mean), crime*log(mean), and location*crime*log(mean)). Stepwise elimination of parameters and parameter interactions was carried out until all remaining parameters were significant. A separate analysis of the difference between local and regional scale fluctuation scaling was done by general regression analysis using the log transformed data and a scale categorical variable (0 ≡ local scale, 1 ≡ regional scale). Scale was assigned based on whether the data were obtained from policing neighborhoods (scale = 0) or from constabulary or larger scale (scale = 1).

## Results

### Fluctuation scaling of mortality

**Figure 1:**
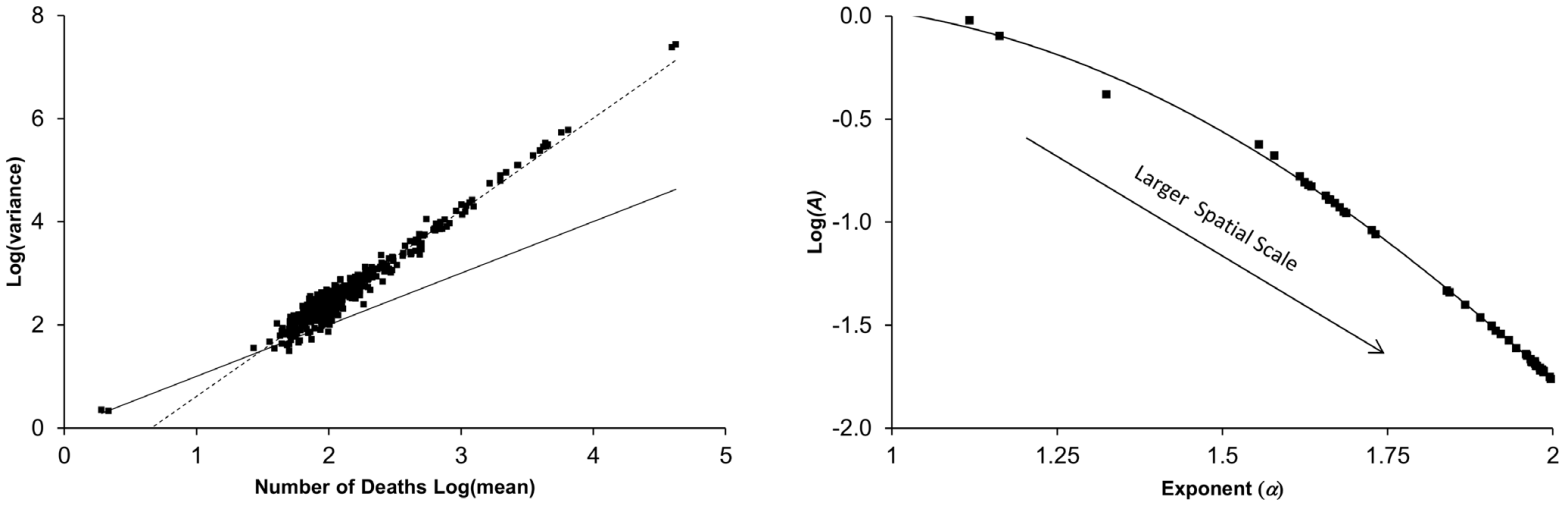
Fluctuation scaling for reported mortality in England and Wales. Left panel: The mean variance plot shows reasonable correspondence to TL (dashed line) with log(*A*) = −1.186±0.041 and *α* = 1.798±0.019. The solid line represents Poisson distributed data with no gain. Multiplication by 10 had no effect on *α*, but changed *A* to 0.1042. Right Panel: The relationship between pre-exponential factor and TL exponent for different data segments in panel on the left. Plot was created by sorting the data by mean value, computing *A* and *α* by linear regression on a 30 point moving segment, and including all values in which *α* was significant with 95% confidence. The arrow represents the approximate direction of the spatial scale. A perfect Poisson process with no gain is a point at (1, 1). The solid line is to guide the eye.

Due to known limitations of UK crime data [34], total mortality data were considered (Data File S1) as a control data set less subject to classification errors or manipulation. The mortality data showed some correspondence to TL (Figure 1, left panel), the exponent was invariant to multiplication, and the pre-exponential factor scaled under multiplication as predicted by equation 2. Inspection of the mean-variance plot revealed clear deviations from TL scaling with lowest mean values approaching the behavior of a Poisson distribution and the highest systematically greater than the predicted values from a single TL fit to the data set. Segmented analysis of the data (Figure 1, right panel) indicated the exponent approached a low scale limit of 1 and a high scale limit of 2 while the pre-exponential ranged from 1 at the low scale limit and ∼0.018 at the higher scale limit.

The high and low scales represent average monthly death counts as low as 2.17 (City of London) and as high as 42,231 (England and Wales). The overall scaling law at low counts appeared to be a Poisson process with no gain. As the mortality increased, temporal clustering became apparent and the TL exponent increased from 1 to 2 beginning at ∼50 deaths (estimated by inspection of Figure 1).

### Fluctuation scaling of Crime

#### Observed behavior within individual policing neighborhoods

**Figure 2:**
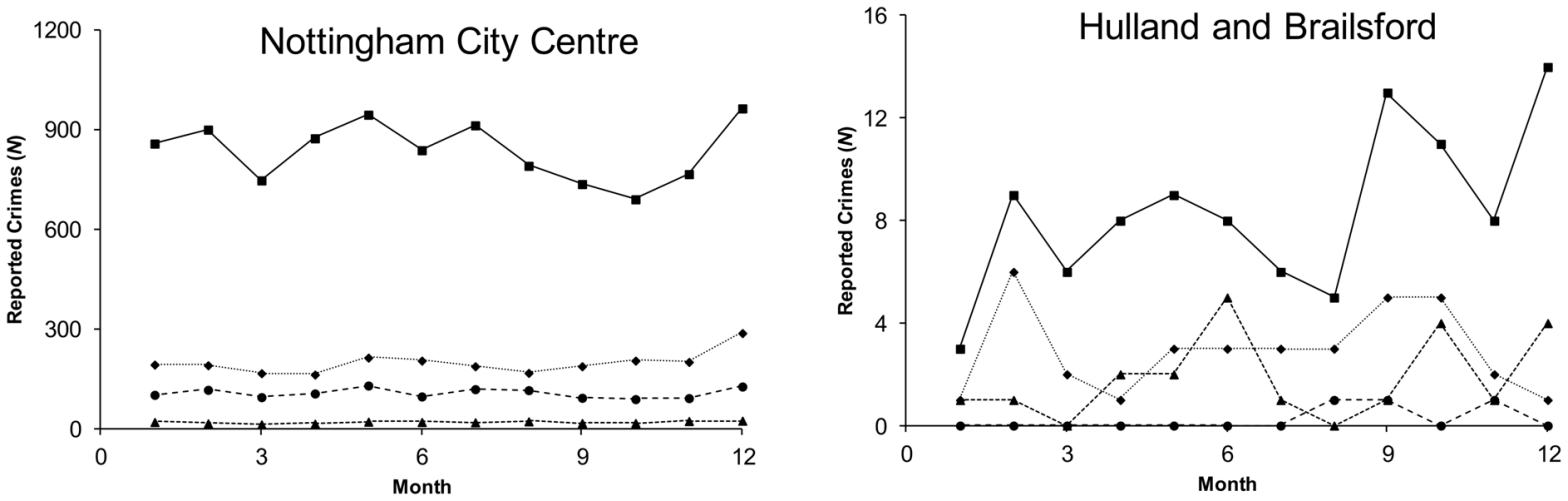
Example data showing reported crimes from high and low crime policing neighborhoods in Nottinghamshire and Derbyshire. Each point represents the number of crimes reported in a one month period. Data is shown for total crime (▪), anti-social behavior (♦), burglary (▴), and violence (•).

The 151 policing neighborhoods within Nottinghamshire and Derbyshire (Data File S2) exhibited total crimes per month ranging from an average of 835 in Nottingham Town Centre to 8 in Hulland and Brailsford (Figure 2). Overall the range of total crime covered slightly over two orders of magnitude with all the data covering 3.5 orders of magnitude. It should be noted that total crime within a policing neighborhood is not an indicator of per capita crime since the population of the policing neighborhoods varied.

#### Local fluctuation scaling in Nottinghamshire and Derbyshire

**Figure 3:**
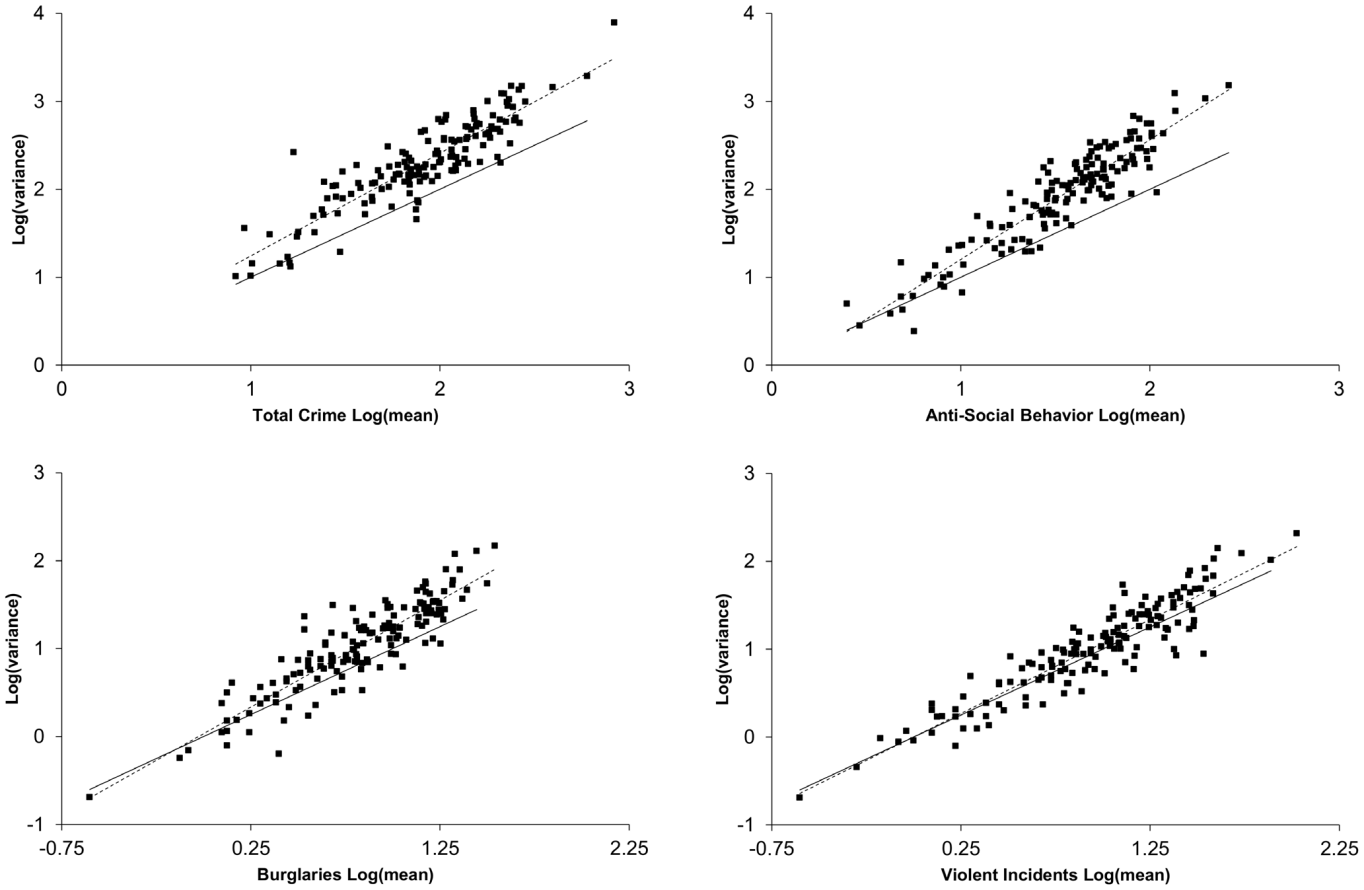
TL plots for the Derbyshire and Nottinghamshire policing neighborhoods. Plots include total crime reports, anti-social behavior incidents, burglaries, and violence. Each point represents the average and variance computed over a 12 month period as represented in Figure 2. Each panel includes the best fit line to the data (dotted line) and a Poisson system having a gain of 1 (solid line). These plots indicate that within a policing region, different crime categories exhibit specific exponents and pre-exponential factors. The data represent 205,857 reported crimes, 84,165 incidents of anti-social behavior, 16,369 burglaries, and 24,759 reports of violence.

Mean-variance plots for crime in Nottinghamshire and Derbyshire (Figure 3) exhibited TL behavior with data covering between 1 and 3 orders of magnitude for individual crime types. In accordance with the behavior of the mortality data under multiplication, in all cases tested the exponent was observed to be invariant under multiplication and the pre-exponential factor scaled in accordance with equation 2. Violence approximated the scaling associated with a Poisson process while all other indicators of crime had exponents considerably greater than one. This suggests greater temporal clustering of the non-violent crimes within the local scale data set. Within the individual TL plots, the exponents and pre-exponential factors varied between the crime categories demonstrating that identical sampling locations (policing neighborhoods) give rise to variable fluctuation scaling relationships for different categories of crime.

#### Global scaling model in Nottinghamshire and Derbyshire

Despite the significance for individual relationships, the presentation by individual crime type (Figure 3) did not address the extent to which *α* and *A* varied significantly by crime type and the regions served by a particular constabulary. A combined data set was used to test this (Table 1) with crime type having a significant effect on *α* (crime*log(mean) in Table 1; p<0.000001) and location having an effect on *A* (location*crime in Table 1; p = 0.00735). The TL exponents for individual crime categories were significantly different from each other with the exception of burglary and anti-social behavior (e.g.: *α*_total_ ≠ *α*_violence_, *α*_burglary_, and *α*_anti-social_; *α*_violence_ ≠ *α*_total_, *α*_burglary_, and *α*_anti-social_; *α*_burglary_ ≠ *α*_violence_ and *α*_total_; *α*_anti-social_ ≠ *α*_violence_ and *α*_total_). There was no evidence values of *α* differed by location, however, *A* differed by location for anti-social behavior (p = 0.014) and burglary (p = 0.019) (Table 2).

**Table 1:** ANOVA Table for the general regression model.

Source	DF	Seq SS	Adj SS	Adj MS	F	P
Regression	7	1810.61	1810.61	258.658	5454.7	0.000000
Log(mean)	1	1805.55	831.89	831.891	17543.4	0.000000
Location*Crime	3	2.60	0.57	0.192	4.0	0.007346
Crime*Log(mean)	3	2.46	2.46	0.819	17.3	0.000000
Error	597	28.31	28.31	0.047		
Lack-of-Fit	535	25.66	25.66	0.048	1.1	0.293128
Pure Error	62	2.65	2.65	0.043		
Total	604	1838.92				

Overall the model explained 98.46% of total variance. The dependent variable is log(variance). In this model, log(mean) is the log_10_ of the average over 12 monthly values for reported crime of a particular type, location*crime indicates how *A* varies for particular crimes between the regions served by the Derbyshire and Nottinghamshire constabularies, and crime*log(mean) indicates the variation in *α* for particular crime types. Note, the significance levels depend on which condition is used as a reference value, but the basic conclusions are unchanged.

**Table 2:** Exponents, pre-exponential factors, and relative gain by location and crime categories for local scale Nottinghamshire and Derbyshire data.

Crime Category	Nottinghamshire				Derbyshire			Notes
	*α*	Log(*A*)	*A*	*G_R_*	*α*	Log(*A*)	*A*	
Total	1.205±0.009	0	1	1	1.205±0.009	0	1	
ASB	1.241±0.017	0.0931±0.0377	1.239±0.108	1.326±0.116	1.241±0.017	0	1	Significant difference between regions in log(*A*) (t = 2.468, p = 0.014).
Burglary	1.292±0.029	−0.0971±0.0414	0.799±0.076	0.728±0.069	1.292±0.029	0	1	Significant difference between regions in log(*A*) (t = −2.345, p = 0.019).
Violence	1.057±0.026	0.0308±0.0422	1.073±0.104	1.078±0.104	1.057±0.026	0	1	No significant difference between regions in log(*A*) (t = 0.730, p = 0.465).*α* is marginally different from 1 (Poisson Distribution) (t = 2.151 p = 0.031).

The multiple categories with *A* = 1 reflects the reference condition selected for the model and the finding that the constant in the regression was not significant and subsequently eliminated from the model.

Application of equations 2–5 and the relative gain method to values of *A* recovered from the regression model provided *G_R_* values subsequently used to correct the number of burglaries and anti-social behavior incidents reported from the two regions. The relative number of burglaries (Nottinghamshire reports/Derbyshire reports) in the two regions based on direct reading of the number of burglaries was 1.15. Application of the *G_R_* adjustment brought the relative number of burglaries closer to 1.57. The raw number ratio for anti-social behavior was 0.75 which became 0.57 after relative gain adjustment. The relative gain parameters suggested a relative under-reporting of anti-social behavior in Derbyshire and under-reporting of burglary in Nottinghamshire. These results should not be interpreted as a finding of fault with the constabularies in the two regions. Rather, the results indicate that direct comparisons of the number or reported crimes between police forces should be treated carefully as the number of crimes resulting in reports may reflect rationally developed public information campaigns, strategies, classification training, and policing priorities in response to local conditions while working with available resources as well as any underlying characteristics of the populations. As noted in the theory section, interpretation of *A* in terms of relative gain (equations 4 and 5), while offering a simple explanation for variation in A, assumes the underlying distributions for these two regions are the same. The values of *G*, *a*, or both could be varying. However, explanations of significant differences in *A* involving *a* changing reinforce the notion that direct comparisons between Police forces should be done with caution. In either case, the data here indicate that reports of violence and total crime between these two forces can be directly compared while anti-social behavior and burglary cannot without recourse to *a* and/or *G*.

#### Local scale manipulation of data

**Figure 4:**
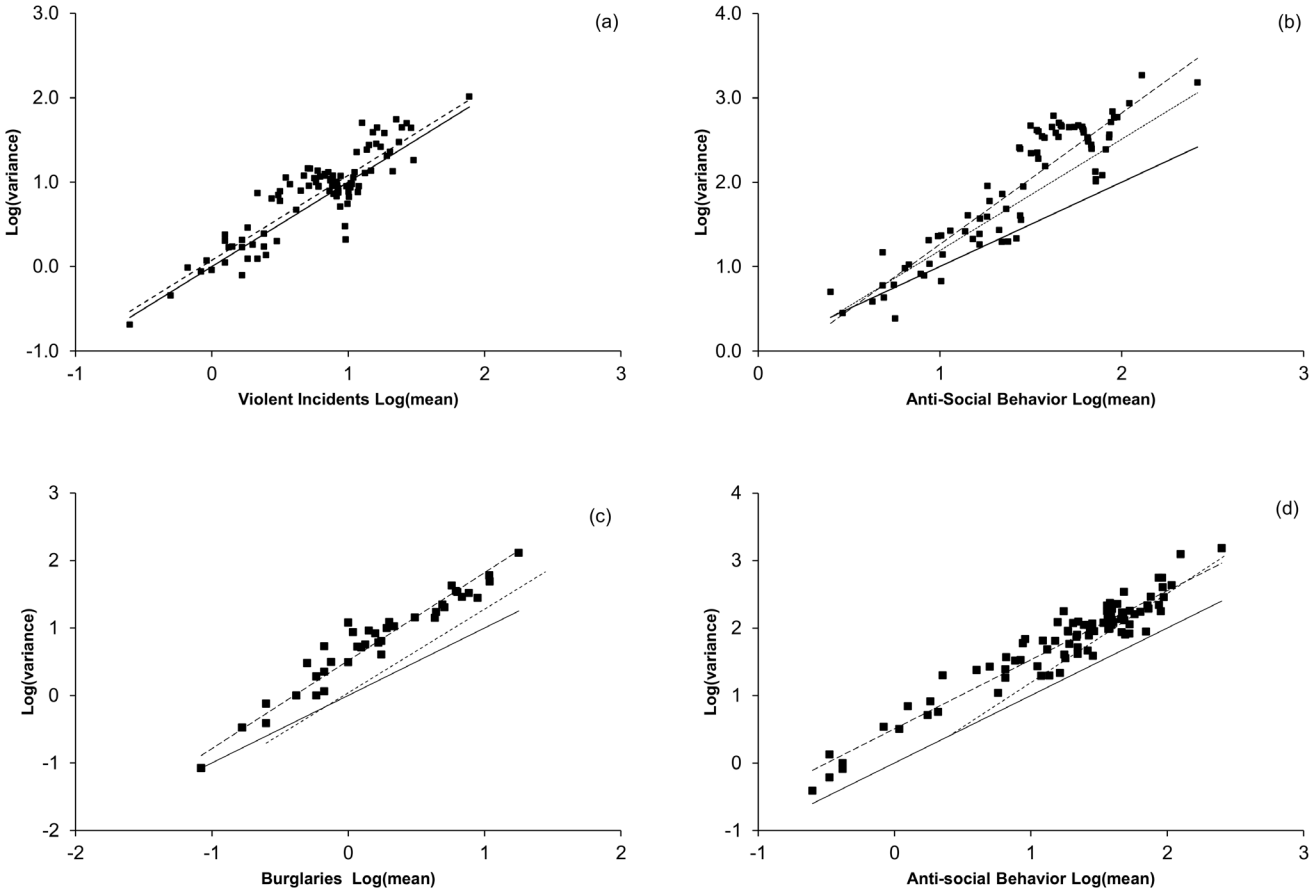
TL plots of the Derbyshire data following data manipulation. Panels a and b illustrate the effects of incentives to reach targets. Panels c and d model the effects of applying a threshold to the reports. Panel a shows data subjected to an incentive to reach 10 reports a month operating above 5 reports. This model consists of reports being “snapped” to 10 once 5 reports are exceeded. Note the gap in the results beginning near 5, the level variance near 10 and subsequent drop in variance at 10. TL fit (long dashed line) is similar to that obtained from the un-manipulated data. Panel b illustrates the impact of an incentive to reach 75 reports with a width of 35 reports. TL fits show how the manipulation results in an increase in exponent and a decrease in pre-exponential factor. Note the gaps in the results beginning near the lower end of the incentive zone, the flattening of variance between the bottom of the incentive zone and the target value, and subsequent drop in variance at 75. These features give clues about the “strength” of the incentive and the target value. Panels c and d are TL plots for data to which a threshold of 10 crimes must be exceeded before a crime is reported (solid squares and long dashed line) in comparison to the original TL fit (dotted line). The solid lines are the behavior of a Poisson distribution with no gain.

To test the effects of simple manipulations of the data set (Data File S2) the Derbyshire neighborhood data were subjected to targets (Figure 4a & 4b), thresholds (Figure 4c & 4d), and multiplication. In all cases, multiplication changed the pre-exponential factor only as described by equation 2. Targets were simulated as a zone over which the data snapped to a target value. For example, if a target was 100 and the zone width 10, then once 91 reports were reached the value would be set to 100. This essentially states that the variance becomes less dependent on the number of reports over a defined interval. Thresholds were modeled by requiring that a minimum number of crimes of a particular type must occur before the first report is generated. In this model, if the threshold is 10, 11 incidents result in 1 report being logged and all subsequent incidents occurring within a month increasing the report count by one. Both types of manipulation generated changes in the TL behavior. Target driven behavior resulted in more constant variance followed by a marked dip in the TL plot which resulted, in some cases, in changed exponents. This feature gives indications about the strength of the incentive and the target value. Application of a threshold resulted in excess variance, a large adjustment of the pre-exponential factor, and a tendency to show concave downward curvature in the TL plot. Although these three models are simplistic, they demonstrate that some manipulations can be detected and that different types of manipulation have different effects on the fluctuation scaling relationships.

### Regional fluctuation scaling of total crime, violence, drug crime, anti-social behavior, burglary, criminal damage and arson, and other crime

**Figure 5:**
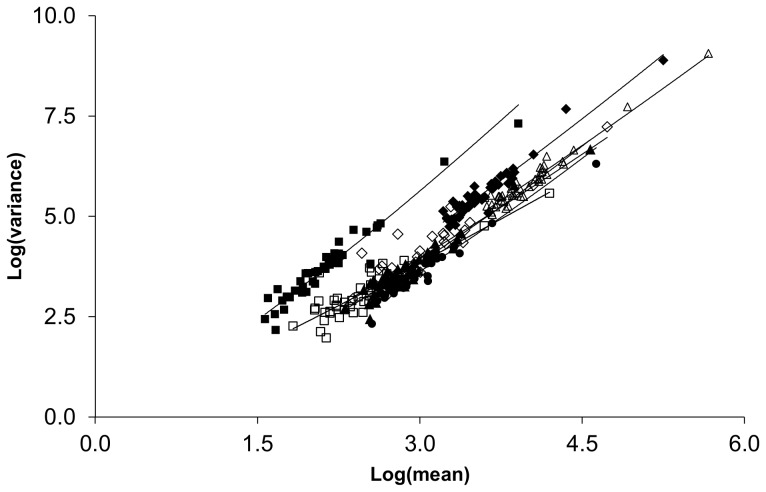
Regional and country scale TL plots for total (open triangle, Log(*A*) = −1.62±0.27, *α* = 1.871±0.066), violence (open diamonds, Log(*A*) = −1.16±0.37, *α* = 1.72±0.12), drugs (open squares, Log(*A*) = −0.42±0.28, *α* = 1.43±0.11), anti-social behavior (filled diamonds, Log(*A*) = −1.70±0.30, *α* = 2.033±0.084, burglary (filled triangles, Log(*A*) = −1.44±0.23, *α* = 1.773±0.078), criminal damage and arson (filled circles, Log(*A*) = −1.57±0.22, *α* = 1.736±0.075), and other crime (filled squares, Log(*A*) = −0.72±0.18, *α* = 2.094±0.081). The data for City of London were classified as local scale. The highest point is country wide aggregation of 43 constabularies.

Regional and country scale (Data File S3) TL plots tended to exhibit greater exponents and smaller pre-exponential factors than did the local data (Figure 5). Across the categories, the maximum exponents appeared to be values near 2 (observed in the case of antisocial behavior and “other” crime). The remaining categories did not reach this value when the scale included all of England, Wales, and Northern Ireland. Within the crime categories studied, “other” crime exhibited strikingly different behavior than the others. The TL plot was characterized by an exponent near 2 observed for relatively few reported crimes combined with a large pre-exponential factor. The reason for this is unclear; however, the “other” category within the UK includes a large number of crimes of deceit, including such things as forgery, fraud, false documents, perjury, tax evasion, and related activities. These crimes may be more fundamentally clustered in nature and hence different from other categories of crime. This is, however, a somewhat simplified view of the category which also includes such things as health and safety offences and dangerous driving. Further work is needed to look at the offences composing the “other” category to better understand the unique characteristics of this grouping.

**Figure 6:**
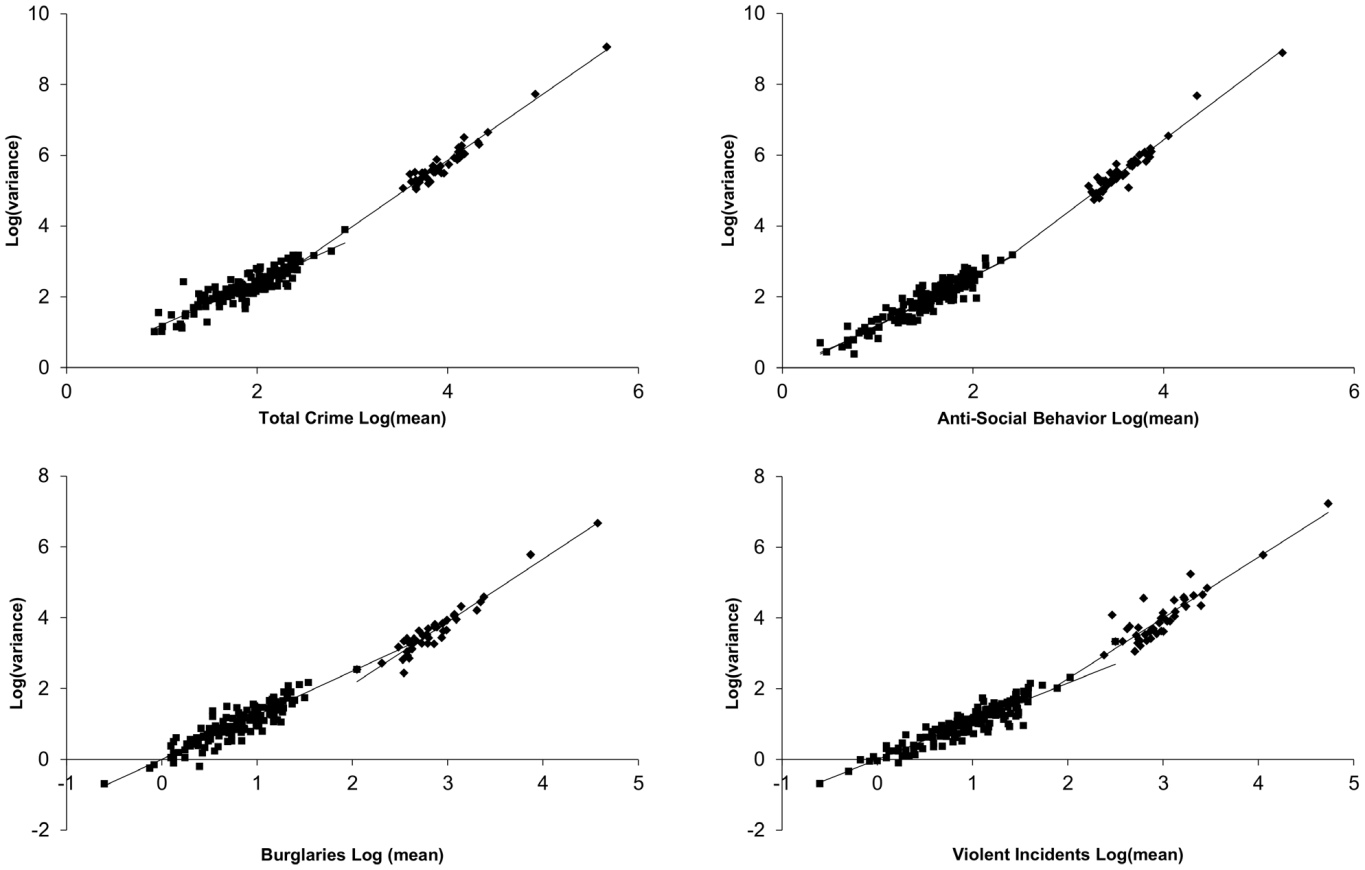
Fluctuation scaling relationships for total crime, anti-social behavior, burglary, and violence spanning local (filled squares), regional and country scales (filled diamonds). Intersection points between the TL relationships for the two scales occurred at 2.44±0.25 (275), 2.30±0.27 (200), 2.72±0.24 (526), and 1.79±0.27 (61) events in log units with number units in parentheses for total crime, anti-social behavior, burglary, and violence, respectively. The highest point in each panel is country wide aggregation of 43 constabularies.

Combined fluctuation scaling plots of local and regional data sets accentuate the extent to which fluctuations across all levels of spatial aggregation deviate from a single TL relationship (Figure 6). As seen in the mortality data, the overall fluctuation scaling relationship could not be explained by a single TL relationship. To test the significance of the difference in behavior at high and low scale, the data were placed in categories of high (constabulary or greater scale) and low (local neighborhoods) scale for statistical tests. In all cases, the high and low scale data exhibited significantly different (p< 0.0001) values for *a* and *α*. It should be noted that a quadratic fit (also highly significant) may also be used to demonstrate that a single TL relationship is insufficient to explain the data at all scales. The intersection of the two TL relationships provides an approximate lower bound on the number of crimes in an observing region before the higher scale behavior can be observed. Using the intersections to define this lower boundary and using it as a proxy for a boundary between regional and local scale, burglary required the largest number of crimes, 526, while violence was roughly an order of magnitude lower, 61.

### Relationship between exponent and pre-exponential factors

**Figure 7:**
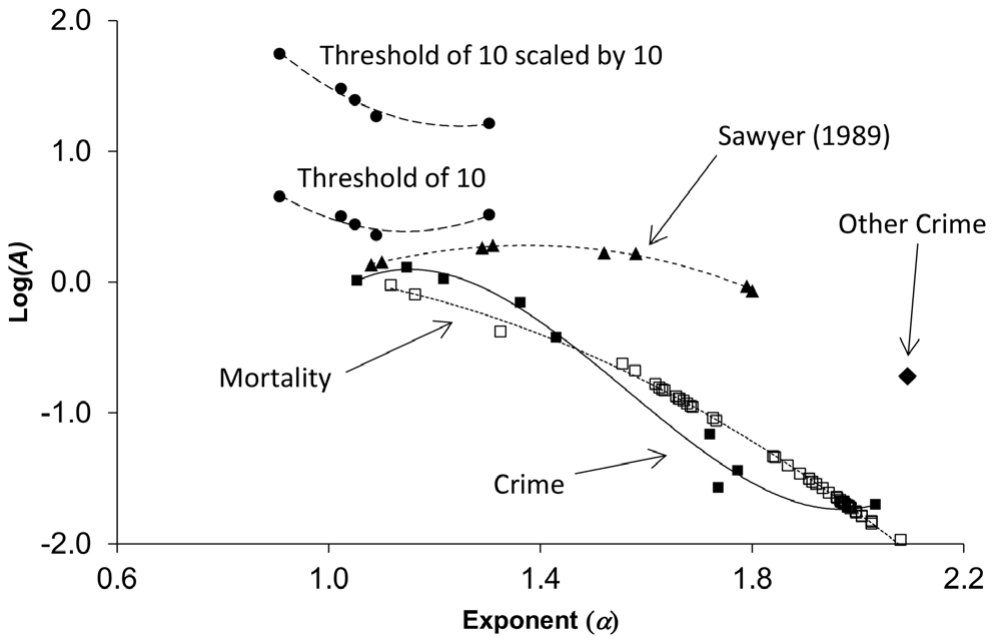
The relationship between *α* and *A* for the crime (filled squares) and mortality (open squares) data, Sawyer’s quadrat size simulations (filled triangles), and data subjected to a threshold and rescaled by a factor (filled circles). Excepting “other” crime (filled diamond), the crime categories and mortality data follow clear trends. “Other” crime appears to be fundamentally different from the remaining crime categories and mortality both in terms of its fluctuation scaling (Figure 5) and the observed values of *α* and *A*.

To better understand the relationship between the pre-exponential factor and the exponent of a TL relationship, these parameters were aggregated over the data sets (Figure 7). This indicates that although the observed TL coefficients vary with the scale as found previously by Sawyer, the crime and mortality data clearly show different behavior than predicted by the hierarchical aggregation model used in the earlier study (with the possible exception of the “other” crime category). It is also noteworthy that the variable sampling procedures compared by Sawyer had some effect on the values of *A* and *α*, but are confined to the same trajectory. Application of a threshold and rescaling by multiplication had the effect of moving the trajectory of *A* and *α* up the co-ordinate system. This suggests, that once well understood through repeated investigations, many types of data manipulation should be observable as excess gain, excess variance, and incommensurate change in the two parameters of the scaling law.

## Discussion

This work establishes that crime reports follow TL at local scales. Violence was only slightly above apparent randomness in its local scale behavior. However, on larger scales it shows more complex behavior. This observation is of interest relative to what is known from population scaling studies of cities. For example, the exponent for serious crime scaling with population (1.18) in US cities indicates crime accelerates with city population [5]. Many other studies investigating serious crime and homicides with urban scaling have found similar results [2], [3], [4], [35]. There is evidence that population scaling laws vary across countries [2]. The well-established observation that serious crime and homicide accelerate in urban areas might indicate a tendency for population centers (e.g. the Nottingham Town Centre and Derby City neighborhoods) to deviate from the more rural areas in violence scaling. The latter was not observable in our data. What is less clear is the extent to which population and fluctuation scaling report on similar processes. Future studies should seek to decouple population scaling from fluctuation scaling. One approach to this might be to revisit the population scaling studies by breaking them down to neighborhoods within cities and see if the scaling laws are a characteristic of the population center as a whole or whether it contains spatially dependent structure.

The data reported here demonstrate that anti-social behavior shows temporal clustering and confirms previous work on clustering of burglaries. Prior work has made clear that incidents of burglary cluster spatially and attempts have been made to understand this clustering behavior using foraging theory [36]. Recent work in China has confirmed the observation that burglary correlates with past history in an area [37]. In the present work, the non-Poisson scaling exponent found in the case of burglary reinforces the notion of clustering and correlation with prior history within local regions. This is fundamentally different from violence in that the clustering behavior is readily observable down to the smallest of scales in the data set. At local scale, there was no evidence for variation in the burglary scaling law exponent across the two regions studied. The robustness of this exponent on the local scale may be responsible for the widespread understanding that burglaries cluster and most observers are unlikely to find it surprising that anti-social behavior clusters as well. It also suggests that further local scale interventions could disrupt the observed local clustering, driving down the covariance of reported crime.

The nature of the location dependent variation in pre-exponential factors for anti-social behavior and burglary is less clear. We are unaware of a discussion of gain applied to power laws outside of electro-optical studies of photon detectors [23]. Relative gain variations could represent such things as a tendency for one police force to classify clustered burglaries into a single crime for reporting purposes and/or different responses to victimization within the two populations. That relative gain variation was specific to particular crimes (burglary and anti-social behavior) and did not systematically apply to the region served by a single police force supports the view that these were true relative gain variations and not artifacts of the neighborhood size or the transition from local to regional and country scale behavior. Future work should seek a better understanding of gain by looking at more constabularies down to local scales and seeking to understand whether practices within constabularies influence gain. This understanding is important as *A* is an indicator of the comparability of statistics arising from different regions. Direct comparisons are clearly invalid in some instances, but equations 1–5 suggest ways to harmonize crime report comparisons.

The contrast between local and regional scale behavior is of fundamental importance. Epidemiology suggests that TL relationships change in response to vaccination programs [13]. If local level policing, criminal justice, and other public policy initiatives “immunize” communities from crime, then these strategies can be evaluated by looking at the scaling parameters. In the case of violence which appears nearly random, there is little that can be done at the neighborhood level to affect the clusters of violent crime. Clustering is nearly invisible at this level and evaluating a police force or its officers based on the rise or fall of violent crime at local level is doing them a disservice. Disruption of larger scale clustering behavior may be more fruitful, but requires larger scale policies and practices. More can be done at local level for the categories of anti-social behavior, burglary, and total crime. For those categories, clustering exists that is visible at local scale and progress can be observed via local fluctuation scaling.

Changes in crime report numbers have been of considerable policy interest within the area of study. Issues relating to manipulation of numbers have been the subject of Parliamentary Committee hearings and widely covered within the popular press. Fluctuation scaling behavior shows promise as a tool to track and evaluate changes in crime reports over time. Fluctuation scaling is a more robust indicator than numbers alone and with further work should allow more clues to underlying mechanisms of crime to be understood. The results here show statistically defensible variations in the behavior of different types of crimes across local and regional scales and between regions served by different constabularies which should allow better targeted interventions. This allows policy makers to move beyond reported crime numbers to develop policies to change the fluctuation scaling relationships more fundamentally. Much further work is needed to look at this behavior over larger regions and in multiple countries.

## Conclusion

We have shown that crime and mortality show temporal fluctuation scaling which is well approximated by TL over high (Constabulary and Country) and low (policing neighborhood) scales and that the TL parameters vary with crime type and location. These statistics are relatively straightforward to calculate and could be evaluated over shorter time scales with appropriately constructed data sets. This will allow for more timely evaluation of the impact of public policy and use of resources using methodology which is potentially more robust to data collection methods. We anticipate fluctuation scaling will become a routine tool in the study of crime.

## Supporting Information

Data File S1 **Monthly Provisional Mortality data for 2013 as provided by the Office of National Statistics (release date 28/01/2014).**
(XLS)

Data File S2 **Local scale raw monthly crime data for Nottinghamshire and Derbyshire with tabs containing threshold, target snap, and multiplication manipulations.**
(XLSX)

Data File S3 **Regional and country scale raw monthly data for England, Wales, and Northern Ireland.**
(XLSX)
